# Weight management intervention identifies association of decreased DNA methylation age with improved functional age measures in older adults with obesity

**DOI:** 10.1186/s13148-021-01031-7

**Published:** 2021-03-02

**Authors:** Curtis L. Petersen, Brock C. Christensen, John A. Batsis

**Affiliations:** 1 The Dartmouth Institute for Health Policy, Williamson Translational Research Bld, 5., 1 Medical Center Drive, Lebanon, NH 03766 USA; 2 Quantitative Biomedical Sciences Program, Dartmouth, Hanover, NH USA; 3 Department of Epidemiology, Geisel School of Medicine at Dartmouth, Lebanon, NH USA; 4 Department of Molecular and Systems Biology at Dartmouth, Lebanon, NH USA; 5 Department of Medicine, Geisel School of Medicine at Dartmouth, Hanover, NH USA; 6 Section of General Internal Medicine, Dartmouth-Hitchcock Medical Center, Lebanon, NH USA; 7 Division of Geriatric Medicine, School of Medicine, and Department of Nutrition, Gillings School of Global Public Health, University of North Carolina at Chapel Hill, Chapel Hill, NC USA

**Keywords:** DNA methylation, Methylation clock, Methylation age, Functional ability, Aging, Healthy aging, Anti-aging

## Abstract

**Background:**

Assessing functional ability is an important component of understanding healthy aging. Objective measures of functional ability include grip strength, gait speed, sit-to-stand time, and 6-min walk distance. Using samples from a weight loss clinical trial in older adults with obesity, we examined the association between changes in physical function and DNA-methylation-based biological age at baseline and 12 weeks in 16 individuals. Peripheral blood DNA methylation was measured (pre/post) with the Illumina HumanMethylationEPIC array and the Hannum, Horvath, and PhenoAge DNA methylation age clocks were used. Linear regression models adjusted for chronological age and sex tested the relationship between DNA methylation age and grip strength, gait speed, sit-to-stand, and 6-min walk.

**Results:**

Participant mean weight loss was 4.6 kg, and DNA methylation age decreased 0.8, 1.1, and 0.5 years using the Hannum, Horvath, and PhenoAge DNA methylation clocks respectively. Mean grip strength increased 3.2 kg. Decreased Hannum methylation age was significantly associated with increased grip strength (*β* = −0.30, *p* = 0.04), and increased gait speed (*β* = 0.02, *p* = 0.05), in adjusted models. Similarly, decreased methylation age using the PhenoAge clock was associated with significantly increased gait speed (*β* = 0.02, *p* = 0.04). A decrease in Horvath DNA methylation age and increase in physical functional ability did not demonstrate a significant association.

**Conclusions:**

The observed relationship between increased physical functional ability and decreased biological age using DNA methylation clocks demonstrate the potential utility of DNA methylation clocks to assess interventional approaches to improve health in older obese adults.

*Trial registration*: National Institute on Aging (NIA), NCT03104192. Posted April 7, 2017, https://clinicaltrials.gov/ct2/show/NCT03104192

## Background

Between 2007 and 2010, approximately 35% of adults over age 65 in the United states were classified as having obesity placing them at increased risk of functional decline, institutionalization and death [1]. Dietary weight loss interventions improve cardiometabolic variables [2] and physical function [3], while reducing the risk of long-term mortality [4]. In contrast, resistance exercise improves aerobic capacity [5] and muscle strength [6], both key determinants of healthy aging and functional independence [7]. The contribution of accumulated co-morbidity and physical deficits to morbidity and mortality makes understanding the relationship between physical ability and age important. The natural progression from independence in daily activities to disability in older adults during aging can be assessed using various physical function measures (e.g., grip strength, gait speed, 5× sit-to-stand test, and 6-min walk tests). Because age-associated functional phenotypes are not solely dependent on age, the relationship has been hypothesized to be impacted by genomics and environmental exposures [8].

While most measures of physical function or functional age are used in clinical settings, new measures of biological age have been developed using genome-scale measures of the epigenome in healthy human subjects [8, 9]. Epigenetics is understood to be one mechanism that mediates the gene-environment relationship [10]. Measures of biological age through epigenetic measures termed “epigenetic clocks” or “DNA methylation clocks” aim to both predict chronological and biological age. DNA methylation is the addition of a methyl group to a cytosine typically in the context of cytosine-guanine (CpG) dinucleotides, which regulates gene expression and cell lineage specification during development and differentiation. Early studies examining genome-scale DNA methylation identified age-related changes to patterns in DNA methylation in normal human tissues [11]. More recently, high-resolution array-based DNA methylation measures in large numbers of healthy normal human specimens from various tissues has led to the development of DNA methylation clocks to estimate chronological age. These clocks use DNA methylation data from unique sets of a few hundred CpG sites as inputs to predict age [8, 12]. Differences between chronological age and DNA methylation age provide new opportunities to understand biological processes of aging [13].

Since the first DNA methylation age clock developed by Horvath [9] several others have been published including the Hannum age clock and PhenoAge [14, 15]. These clocks differ from each other in the population, tissue types, ages, and outcomes used in their development. Each clock has demonstrated high accuracy in predicting one’s age. Yet, a methylation clocks potential value is in its ability to show the acceleration and deceleration of biological age in contrast to static measures of chronological age as it may be predictive of changing health status, increased risk of morbidity and mortality [8, 16, 17]. Simultaneously exploring a clock in a clinical context may determine whether differences or associations exist and/or are dependent on underlying biological processes being explored [18]. Further, it has not been established if each DNA methylation age clock performs the same in a given clinical or biological context. For instance, Kresovich et al. (2019) examined if DNA methylation age acceleration was associated with breast cancer risk by testing the Horvath, Hannum, and PhenoAge DNA methylation clocks finding that age acceleration in each was associated with increased risk of breast cancer—though there was variation in their association [18]. It is plausible that different methylation clocks require evaluation in different populations and disease states in efforts to be eventually be used in precision medicine.

Previous studies have investigated the relationship between physical function and changes in methylation age [19–21]. To our knowledge, none have examined its impact within the context of a clinical trial with repeated measures, and only one DNA methylation clock (Horvath) was evaluated. Results are mixed with two studies finding an association with grip strength and a single DNA methylation clock, raising the question if phenotypic measures of biological age are associated with any of the methylation based biological age markers. It is also unclear if such an association is dependent on the specific combination of functional measure and DNA methylation age used, or the time scale over which they would have to be measured to observe an association. We investigated the relationship of physical function with changes in epigenetic biological age using three commonly used DNA methylation-based clocks over a 12-week diet-exercise weight-loss intervention in older adults with obesity. Our exploratory findings provide a formative step of understanding each clock’s clinical relevance within the context of a health promotion intervention.

## Results

A total of 32 pre/post peripheral blood samples were collected which had paired physical functioning data from 16 individuals. Mean participant age was 73 ± 5.7 years, and 14 (87%) were female. At baseline there was a difference in grip strength between those included and excluded (*p* = 0.04). There were no observed differences in all other baseline participant characteristics between the 16 individuals that had pre-post blood collected compared to the 12 individuals that were missing at least one blood sample collection (Additional file 1: Table 1). The samples used in this analysis were chosen due to having complete blood samples and not because of any other baseline characteristic in the larger sample.

**Table 1:** Change between pre- and post-measurements

	Baseline	Week 12	Difference	*p* value
Age years, mean ± SD	73.50 ± 5.72			
Sex				
Female	14 (87.5%)			
Male	2 (12.5%)			
Body Measures, mean ± SD				
Weight (kg)	95.7 ± 21.3	91.5 ± 22.1	− 4.2 ± 3.7	< 0.001
Body Mass Index (kg/m^2^)	36.2 ± 7.0	34.6 ± 7.4	− 1.6 ± 1.4	< 0.001
Fat Mass (%)	49.0 ± 5.0	48.0 ± 5.5	− 0.9 ± 2.4	0.13
Visceral Fat mass, (L)	4.0 ± 1.8	3.6 ± 2.2	− 0.4 ± 1.0	0.15
Skeletal muscle mass (kg)	48.8 ± 15.9	47.5 ± 15.0	− 1.3 ± 2.3	0.04
Appendicular lean mass (kg)	12.3 ± 4.0	12.1 ± 3.8	− 0.3 ± 0.7	0.11
Functional measures, mean ± SD				
Grip strength (kg)	18.7 ± 5.9	21.9 ± 6.6	3.2 ± 4.1	0.008
Gait speed (sec)	1.1 ± 0.3	1.1 ± 0.25	0.02 ± 0.2	0.69
Sit-to-stand (sec)	10.0 ± 2.7	7.6 ± 1.8	− 2.4 ± 2.2	< 0.001
Six-minute walk (m)	406.4 ± 101.1	448.6 ± 82.12	29.3 ± 34.6	0.006
Immune Cell Proportions, mean ± SD				
CD8T	0.06 ± 0.02	0.06 ± 0.02	0.00 ± 0.01	0.16
CD4T	0.18 ± 0.03	0.18 ± 0.03	− 0.00 ± 0.02	0.81
Natural Killer Cells	0.05 ± 0.02	0.05 ± 0.02	0.00 ± 0.01	0.4
B Cells	0.05 ± 0.02	0.05 ± 0.02	− 0.00 ± 0.01	0.76
Monocyte	0.08 ± 0.02	0.07 ± 0.02	− 0.00 ± 0.01	0.06
Neutrophils	0.62 ± 0.05	0.62 ± 0.05	− 0.00 ± 0.03	0.97
Methylation age clocks, mean ± SD				
Hannum	79.4 ± 6.8	78.6 ± 6.6	− 0.8 ± 4.8	0.51
Horvath	79.3 ± 6.8	78.2 ± 6.4	− 1.1 ± 2.8	0.14
PhenoAge	62.7 ± 6.4	62.3 ± 7.0	− 0.5 ± 4.1	0.64

Table 1 shows summary statistics for participant characteristics and physical function measures at both time points. Over the 12-week study period, mean weight-loss was 4.2 ± 3.7 kg; 8 participants experienced a ≥ 5% weight loss. There was a significant decrease in weight, body mass index (BMI), and skeletal muscle mass in patients from baseline to follow up. Participants showed significantly improved grip strength (21.2 ± 28.0%, *p* < 0.01), sit-to-stand times (− 20.7 ± 19.2%, *p* < 0.001), and 6-min walk test (8.5 ± 10.5%, *p* < 0.006). We did not observe significant changes in cell type proportions between baseline and follow-up. The reduction in pre post DNA methylation age in the Hannum, Horvath, and PhenoAge clocks did not reach statistical significance.

The unadjusted association between each physical function measure (grip strength, gait speed, sit-to-stand, and 6-min walk) and DNA methylation age clock (Hannum, Horvath, and PhenoAge) were all directionally consistent within each outcome (Table 2). Lower chronological age was associated with improved grip strength, sit-to-stand, and the 6-min walk test. Men had significantly improved grip strength, sit-to-stand time. Our exploratory, unadjusted models demonstrated significance with the PhenoAge and grip strength; we did not show any significant associations with gait speed, sit-to-stand or 6-min walk tests.

**Table 2:** Unadjusted associations of age, sex, and change in methylation age clocks with change in grip strength, gait speed, sit-to-stand, and 6-min walk distance functional measures

	Grip strength		Gait speed		Sit-to-stand		Six-minute walk	
	*β* (95%CI)	*p* value	*β* (95%CI)	*p* value	*β* (95%CI)	*p* value	*β* (95%CI)	*p* value
Age	− 0.44 (− 0.77 to 0.11)	0.013	− 0.01 (− 0.03 to 0.01)	0.192	0.22 (0.03 to 0.40)	0.027	− 3.50 (− 6.60 to 0.40)	0.030
Sex								
Female								
Male	8.12 (2.96 to 13.28)	0.005	0.14 (− 0.15 to 0.43)	0.326	− 4.03 (− 6.97 to 1.08)	0.011	47.14 (− 4.58 to 98.86)	0.071
Methylation age clocks								
Hannum	− 0.34 (− 0.89 to 0.21)	0.201	0.02 (− 0.01 to 0.04)	0.184	0.13 (− 0.17 to 0.44)	0.368	− 0.85 (− 5.87 to 4.16)	0.719
Horvath	− 0.15 (− 0.99 to 0.69)	0.704	0.02 (− 0.01 to 0.06)	0.178	0.24 (− 0.19 to 0.68)	0.245	− 2.21 (− 9.92 to 5.50)	0.547
PhenoAge	− 0.45 (− 0.87 to 0.02)	0.041	0.01 (− 0.00 to 0.03)	0.127	0.12 (− 0.14 to 0.37)	0.351	− 2.24 (− 6.26 to 1.78)	0.249

Our primary goal was to evaluate the association of functional measures with DNA methylation clocks. To account for potential confounding by age and sex we used adjusted linear regression models, which generally demonstrated a significant association between improvement in physical function a decreased DNA methylation age. Specifically, Table 3 shows the main results of these models by physical functional ability measure and DNA methylation age clock. Increasing grip strength was significantly associated with decreasing Hannum DNA methylation clock (*β* = −0.30, *p* = 0.041), and though not statistically significant, the direction of effect was consistent with decreased DNA methylation age for the Horvath and PhenoAge clocks. Decreasing gait time was significantly associated with decreasing DNA methylation age from the Hannum (*β* = 0.02, *p* = 0.049) and PhenoAge (*β* = 0.02, *p* = 0.039) clocks, and again the direction of effect was consistent with decreasing Horvath methylation age (*β* = 0.02, *p* = 0.134). Decreasing sit-to-stand time and increasing 6-min-walk distance demonstrated no significant association with any decreasing methylation age.

**Table 3:** Adjusted associations between measures of change in physical function and change in DNA methylation age

Methylation age clocks	Grip strength		Gait speed		Sit-to-stand		Six-minute walk	
	*β* (95%CI)	*p* value	*β* (95%CI)	*p* value	*β* (95%CI)	*p* value	*β* (95%CI)	*p* value
Hannum	− 0.30 (− 0.59 to − 0.01)	0.041	0.02 (0.00 to 0.04)	0.049	0.04 (− 0.17 to 0.24)	0.700	− 1.52 (− 5.06 to 2.02)	0.364
Horvath	− 0.05 (− 0.62 to 0.52)	0.862	0.02 (− 0.01 to 0.06)	0.134	0.19 (− 0.13 to 0.52)	0.215	− 2.61 (− 9.02 to 3.80)	0.389
PhenoAge	− 0.10 (− 0.51 to 0.31)	0.605	0.02 (0.00 to 0.05)	0.039	0.01 (− 0.25 to 0.26)	0.950	0.84 (− 3.58 to 5.25)	0.685

Adjusted models for the association of each DNA methylation-based age clock and each functional measure controlling for chronological age and sex. All effects are in the same relative direction for each methylation age clock and each functional measure, except the association of PhenoAge and the six-min walk

## Discussion

Our findings suggest that improved grip strength and gait speed was associated with decreased DNA-methylation age in older adults with obesity participating in a weight loss trial. These findings potentially link physical function in older adults with biological age.

Our results extend the findings of previous studies that only used a single DNA methylation age clock. Marioni et al. [21] examined the association between grip strength and the Horvath Clock both cross-sectionally, and longitudinally at a 6-year follow-up timepoint. These authors observed a correlation between increasing grip strength and lower methylation age in older adults aged 70 in the cross-sectional analysis, but not in their longitudinal analysis [21]. Simpkin et al. (2017) found a significant association between Horvath Clock age acceleration and change in grip strength 7–10 years later in women and no association with knee extension strength and walking speed [19]. These results were corroborated in a cross-sectional study of twins aged 54–72 years measuring grip strength and the Horvath Clock, though there was no association with knee extension strength, and walking speed [20]. Such results are mixed because of the heterogeneity between the study populations and the functional measures used. The heterogeneity may also be due to differential effects of sub-populations and unmeasured factors such as fitness or body composition.

The multiple DNA methylation clocks that have been developed are highly correlated with age and each other [18]. The difference between chronological age and DNA methylation age represents an age acceleration or deceleration. Though correlated, DNA methylation age values vary slightly from one clock to another as they use different loci across the genome to infer age. Each unique mixture of loci may be differentially associated with environmental or behavioral factors. Simultaneous investigation of results from multiple DNA methylation clocks informs how they might be consistent or differ in specific settings.

We observed that a decrease in Hannum and PhenoAge DNA methylation ages were associated with improvement in grip strength and gait speed functional measures over a short time period in this intervention study. Prior work has examined changes over years; whether such changes can be observed in shorter time frame is unclear. All the statistical models examined, except one, demonstrated consistent direction of effect—increasing physical function is associated with decreasing DNA methylation age, yet we were not able to demonstrate a significant association. While we acknowledge that our study has relatively limited sample size, these findings suggest the need to continue the study of DNA methylation age in short-term interventional studies in larger and more racially and ethnically diverse populations. Because of the limited sample size, we did not conduct locus-by-locus comparisons or any subgroup analysis.

As there are potential sex differences in DNA methylation age clocks and allostatic load [22] the relationship between functional measures and DNA methylation age may be impacted by sex. While allosteric load can be measured in multiple ways there could be a relationship between these and functional measures. Here we adjusted for sex in our models to account for any underlying differences due to sex. Race/ethnicity—another source of variation in DNA methylation was not included in the model as all participants were the same identifying as White, non-Hispanics. Because of the sex and race homogeneity in the study participants our results should be validated in a more diverse cohort.

While the results demonstrated consistent direction of effect, they were not all significant. These discrepancies could be due to a combination of sample size and aspects of biological aging in older adults not yet fully explored. The variation in DNA-methylation age clocks within different older adult sub-populations have also not been examined. The differences between each clock could be due to differences in study population age distribution and age-associated comorbidities used for the clock’s development. Though developed to be age and tissue agnostic [9], the data suggest that the Horvath DNA methylation clock performs best with samples from those between birth and adolescence [23]. One possible reason that we observed a significant signal from the Hannum and PhenoAge DNA methylation age clocks is that the Hannum clock was developed using blood from only adults [14] and PhenoAge was developed with all-cause mortality as its end point [15]. Our results across clocks are similar with those of Kresovich et al. (2019) where there was consistency in direction of effect and scope of effect size. Additional potential sources of variation in DNA methylation could be driven by the observed change in weight or skeletal muscle, both of which significantly decreased over the study period. Having a relationship with body measures such as weight, and skeletal muscle, may impact how they are associated with physical functional measures. Due to a limited sample size in this study we did not add these variables to our model and their relationship should be examined in studied with larger power. The small cohort and its non-random selection based on having biological sample could impact cohort co-variate distributions. Additionally, as a pilot study we did not have a control arm which should be examined in future studies. As we did not observe significant changes in immune cell type proportions within subjects from baseline to follow up, it is unlikely that cell type is driving associations of age acceleration with functional ability. The change in DNA methylation age and future research should validate these findings with a larger more diverse cohort using multiple DNA methylation age clocks and measures of functional ability.

All 16 study participants completed the 12-week weight loss intervention. The intervention which included biweekly group exercise sessions and dietary counseling demonstrates the feasibility of positively impacting physical functional ability and DNA methylation age. As this study’s subjects had relatively low baseline functional ability we demonstrate that those with the greatest need can impact their health through clinical intervention. Further examination of methylation change at specific loci should be considered in a larger cohort to examine the role that DNA methylation might play in mediating increased functional ability. It is possible that positive lifestyle changes in older adults lead to changes in DNA methylation which then impact downstream transcription and translation that lead to increased functional ability. Such changes in DNA methylation could be captured by some or all DNA methylation age clocks making them a valuable measure in lifestyle change interventions.

Finally as BMI has been associated with aging phenotypes [24] and altered DNA methylation [25] by limiting to adults with obesity in this study, methylation-based biological age may be modifiable through clinical interventions. As there was no association between change in DNA methylation age and change in BMI or muscle mass measures (Additional file 1: Table 2), the association with physical functional measures indicates some clinical meaning with age. As there is evidence of differential DNA methylation in skeletal muscle tissue between young and old participants [26] and association between DNA methylation and physiological traits in skeletal muscle [27] examining the change of DNA methylation age in the muscle of older adults as they increase their activity could yield important understanding in how muscle changes with age and how physical exercise and lifestyle can impact it. Specifically, as differential methylation of genes in the HOX family have been associated with physical activity and age in muscle cells it would be important to examine if this relationship is found in older adults who increase their physical functional ability [27]. Physical function measures are clinically relevant in ascertaining an individual’s spectrum of health, ability to fully function, and whether they are at risk for disability. As individuals included in the study had a low baseline grip strength (as compared to those non-included due to missing blood sample), the associated change in DNA methylation age suggests that DNA methylation age could feasibility be used as a measure for individuals that need to improve their functional ability. As older adults with a higher grip strength at baseline (e.g., have higher function), are likely to respond less to the intervention it is reasonable to believe there is likely a ceiling effect of the change in DNA methylation age. The association of DNA methylation age clocks with these measures suggests that it is not measuring a single physical attribute such as BMI or muscle mass, but multiple attributes related to functional ability. Additional work is needed on larger cohorts to evaluate measures of phenotypic aging and their association with methylation-based biological age. Specifically, because of the complex interaction between muscle and fat leading to sarcopenic obesity, more work should be done examining the association between DNA methylation age and this geriatric syndrome. Similar results have been reported in examining the association between DNA methylation age clocks and blood biomarkers, BMI, and demographic information in a young adults with diabetes—that is there was variation in the association of each clock and each clinical measure [28]. Our study suggests that DNA methylation age has clinical relevance as a biomarker for physical function. The results demonstrate an association between increasing physical functional ability measured in the clinic and decreasing methylation based biological age in blood.

## Conclusions

We observe a relationship between increased physical functional ability and decreased biological age using DNA methylation clocks. These results demonstrate the potential utility of DNA methylation clocks to assess interventional approaches to improve health in older obese adults.

## Methods

### Study design

Participants completed a 12-week multi-component weight-loss intervention consisting of dietary counseling and aerobic/resistance exercise activity in older adults with obesity. All participants were aged ≥ 65 years with a body mass index > 30 kg/m^2^. Details of the design, recruitment, retention and preliminary effectiveness results are previously described [29]. Briefly, individuals were recruited from the Dartmouth Center for Health and Aging, a community aging resource center and local primary care practices. Older adults, aged > 65 years, with a body mass index > 30 kg/m^2^ who were willing to participate in the weight loss intervention were included. We excluded participants who had an electronic medical record-based diagnosis that could impact participation in the clinical trial (dementia, uncontrolled psychiatric illness, bariatric surgery, advanced systemic illness, life-threatening or end-of-life illness). Those participating in other weight-loss endeavors, taking obesogenic medications, or an intentional weight loss of ≥ 5% in the past six months were also excluded. Participants answered a demographic questionnaire and completed physical functional measures (see below). Body measures, including height and weight (using an A + D scale) were assessed using a trained research assistant. Fat mass, appendicular lean mass, and skeletal muscle mass were measured using a Seca 512 bioelectrical impedance analyzer.

As part of the clinical trial, adults were asked to provide blood samples at baseline (prior to the intervention) and at a 12-week follow-up. During the 12-week intervention individuals participated in weekly dietary counseling sessions and aerobic/resistance exercise activity sessions.

All participants provided written consent for the study, which was approved by the Committee for the Protection of Human Subjects at Dartmouth College and the Dartmouth-Hitchcock Institutional Review Board and registered on clinicaltrials.gov (NCT03104192).

### Physical function measures

All measures were assessed at baseline and at the conclusion of the intervention. Grip strength was measured as a surrogate marker to upper extremity strength known to predict mobility and disability [30–32]. Grip strength was recorded as the mean of three measures of the participant’s dominant hand using a JAMAR Plus dynamometer with a 30 s rest between each trial, while sitting comfortably in a chair, with their arm at 90°. Gait speed is a simple clinical measure reflecting health and functional status [33] that predicts falls, disability, and mortality [34]. This measure was assessed using a 4-m course, with a 1-m “walk-in” phase before and after the timed segment. The five-times sit-to-stand test is a strong correlate of quadriceps strength and leg press [35, 36]. This test was measured as the amount of time that a participant took to go from a sitting position in the edge of a chair to standing without using their arms a total of five times in a row. The 6-min-walk test is a measure of submaximal aerobic capacity evaluating the distance that an individual can walk over a 6 min timeframe, and is an established measure of functional ability [32, 37]. All measures were conducted by a trained research assistant in a community-aging center in Lebanon, New Hampshire.

### Methylation data

For this analysis, the 16 individuals analyzed were part of a convenience sample from the original cohort (*n* = 28) based on the availability of both a pre and a post sample. Because of logistical issues during collection, we resulted in having missing samples, having only a blood sample at a single time point for some individuals. These individuals could not be included in this analysis and their missingness can be considered at random. Peripheral blood was collected and frozen at baseline and 12-week follow up visits. All genomic DNA extraction and bisulfite conversion, using the Zymo EZ-96 DNA Methylation Kit (Zymo Research), were conducted at the same time. DNA methylation levels were measured at > 850,000 CpG sites across the genome using the Illumina EPIC Human Methylation microarray (EPIC array). Hybridization and processing of samples were performed according to the instructions of the manufacturer. Probe intensity data (IDAT files) processed with the minfi [38] version 1.24.0 R package. All samples passed quality assessment and control of having signal intensity significantly higher than background (*p* > 0.05). Probe signals underwent standard background correction and normalized through noob and beta-mixture quantile normalization methods [39, 40].

### Statistical analysis

Descriptive statistics used paired *t*-test for continuous measures when comparing pre-post change. Students *t*-test and chi-squared tests where used to compare baseline characteristics of the study cohort (*n* = 16) and the study population that did not have blood samples (*n* = 12). The Hannum, Horvath, and PhenoAge DNA methylation age clocks [14] were calculated through the ENmix [40] package in Bioconductor. We used linear regression models to examine the association between physical functional ability and DNA methylation-based age. The association for each outcome—grip strength, gait speed, and sit-to-stand time—was calculated first in unadjusted bivariate models. We then used multivariate models with each functional measure outcome and each DNA methylation age clock while adjusting for age at start of study and sex. Cell type proportions were determined for each sample through a modified version of the Houseman method [41] using the FlowSorted.Blood.EPIC package [42]. Differences in cell type proportions were calculated using paired t-tests. All analysis were conducted in R version 3.6 [43] and significance was defined as *α* < 0.05.

## Supplementary Information

**Additional file 1:** Table 1 examines the differences between individuals who had a blood sample collected and those that did not. Additionalanalysis examining the association between change in body measures and DNA methylation age in Table 2.

## Data Availability

The datasets used and/or analyzed during the current study are available from the corresponding author on reasonable request.

## Abbreviations

CpG: Cytosine-guanine dinucleotide
NLR: Neutrophil to lymphocyte ratio
BMI: Body mass index
