# Supplementary material for: Weight management intervention identifies association of decreased DNA methylation age with improved functional age measures in older adults with obesity
Source: Clin Epigenetics. 2021 Mar 2;13:46. doi: 10.1186/s13148-021-01031-7 (PMC7927264; doi:10.1186/s13148-021-01031-7)
Supplement: Supplementary file 1 — Additional file 1: Table 1 examines the differences between individuals who had a blood sample collected and those that did not. Additionalanalysis examining the association between change in body measures and DNA methylation age in Table 2. [file 13148_2021_1031_MOESM1_ESM.docx]

**Supplemental Table 1: Characteristics of those with and without blood samples. As blood samples were not collected from all individuals in the parent study we compare the groups included in this analysis with those that were not.**

|  | **Methylation Data** (N=16) | **No Methylation Data** (N=12) | | **Total** (N=28) | **p value** |
| --- | --- | --- | --- | --- | --- |
| **Age** years, mean ± SD | 73.50±5.72 | 72.17±4.93 | | 72.93±5.34 | 0.52 |
| **Sex** |  |  | |  | 0.39 |
| Female | 14 (87.5%) | 9 (75.0%) | | 23 (82.1%) |  |
| Male | 2 (12.5%) | 3 (25.0%) | | 5 (17.9%) |  |
| **Marital status** | |  |  | |  |
| Married | 7 (43.75%) | 7 (8.33%) | | 14 (50.0%) |  |
| Divorced | 7 (43.75%) | 5 (41.67%) | | 12 (42.86%) |  |
| Widowed | 2 (12.50%) | 0 | | 2 (7.14%) |  |
| **Body Measures**, mean ± SD |  |  | |  |  |
| Weight (kg) | 95.74±21.32 | 101.86±15.46 | | 98.36±18.96 | 0.41 |
| Body Mass Index (kg/m^2^) | 36.19±7.03 | 38.38±4.73 | | 37.13±6.14 | 0.36 |
| Fat Mass (%) | 48.95±4.95 | 48.28±7.14 | | 48.66±5.87 | 0.77 |
| Visceral Fat mass, (L) | 4.00±1.78 | 4.78±1.66 | | 4.33±1.74 | 0.25 |
| Skeletal muscle mass (kg) | 48.80±15.87 | 53.25±10.52 | | 50.70±13.78 | 0.41 |
| Appendicular lean mass (kg) | 12.34±4.00 | 13.52±2.83 | | 12.85±3.53 | 0.39 |
| **Functional measures**, mean ± SD |  |  | |  |  |
| Grip strength (kg) | 18.73±5.93 | 25.05±9.53 | | 21.44±8.17 | 0.04 |
| Gait time (sec) | 1.07±0.25 | 1.23±0.20 | | 1.14±0.24 | 0.08 |
| Sit-to-stand (sec) | 9.99±2.74 | 9.47±3.03 | | 9.77±2.83 | 0.64 |
| Six-minute walk (m) | 406.44±101.07 | 405.06±74.94 | | 405.85±89.24 | 0.97 |

**Supplemental Table 2: Adjusted associations between measures of change in body measures and change in DNA methylation age.** Adjusted models for the association of each DNA methylation-based age clock and each body measure controlling for chronological age and sex.

|  | **Appendicular lean mass (kg)** | | **Fat mass (kg)** | | **Skeletal muscle mass (kg)** | | **Visceral Fat mass, (L)** | | **Weight (kg)** | |
| --- | --- | --- | --- | --- | --- | --- | --- | --- | --- | --- |
| **Methylation age clocks** | **β (95%CI)** | **p-value** | **β (95%CI)** | **p-value** | **β (95%CI)** | **p-value** | **β (95%CI)** | **p-value** | **β (95%CI)** | **p-value** |
| Hannum | -2.25 (-6.31-1.81) | 0.25 | -0.28 (-1.20-0.65) | 0.52 | -0.81 (-1.95-0.34) | 0.15 | -0.55 (-3.58-2.48) | 0.7 | -0.35 (-1.17-0.47) | 0.37 |
| Horvath | -0.81 (-3.36-1.74) | 0.50 | 0.24 (-0.30-0.79) | 0.35 | -0.31 (-1.04-0.43) | 0.38 | 0.30 (-1.53-2.14) | 0.72 | 0.08 (-0.43-0.59) | 0.73 |
| PhenoAge | -1.17 (-4.64-2.29) | 0.47 | 0.01 (-0.76-0.78) | 0.98 | -0.30 (-1.32-0.71) | 0.53 | 1.05 (-1.38-3.47) | 0.37 | -0.11 (-0.80-0.59) | 0.75 |
